# Edible Medicinal Guava Fruit (*Psidium guajava* L.) Are a Source of Anti-Biofilm Compounds against *Pseudomonas aeruginosa*

**DOI:** 10.3390/plants13081122

**Published:** 2024-04-17

**Authors:** Christian Emmanuel Mahavy, Andriantsihoarana Jonathan Razanatseheno, Adeline Mol, Jeremie Ngezahayo, Pierre Duez, Mondher El Jaziri, Marie Baucher, Tsiry Rasamiravaka

**Affiliations:** 1 Laboratory of Biotechnology and Microbiology, University of Antananarivo, BP 906, Antananarivo 101, Madagascar; mitsinjomahavy@gmail.com (C.E.M.); tsihoaranajo@gmail.com (A.J.R.); 2 Laboratory of Plant Biotechnology, Université Libre de Bruxelles, B-1050 Brussels, Belgium; adeline.mol@ulb.ac.be (A.M.); jaziri@ulb.ac.be (M.E.J.); marie.baucher@ulb.ac.be (M.B.); 3 Centre de Recherche en Sciences Naturelles et de l’Environnement (CRSNE), Université du Burundi, Bujumbura BP 2700, Burundi; jeremie.ngezahayo@ub.edu.bi; 4 Unit of Therapeutic Chemistry and Pharmacognosy, University of Mons, B-7000 Mons, Belgium; pierre.duez@umons.ac.be

**Keywords:** β-sitosterol-β-D-glucoside, biofilm, edible medicinal plants, lycopene, *Pseudomonas aeruginosa*, *Psidium guajava*

## Abstract

*Psidium guajava* is one of the most common edible medicinal plants frequently used in Malagasy traditional medicine to treat gastrointestinal infections. In order to evaluate their probable antibacterial activities, three organic extracts (successive extractions by hexane, dichloromethane, and ethanol) of ripe guava fruits were assessed for their bactericidal and anti-virulence properties against *P. aeruginosa* PAO1. Although these three extracts have shown no direct antibacterial activity (MIC of 1000 µg/mL) and, at the non-bactericidal concentration of 100 µg/mL, no impact on the production of major *P. aeruginosa* PAO1 virulence factors (pyocyanin and rhamnolipids), the hexane and dichloromethane extracts showed significant anti-biofilm properties and the dichloromethane extract disrupted the *P. aeruginosa* PAO1 swarming motility. Bioguided fractionation of the dichloromethane extract led to the isolation and identification of lycopene and β-sitosterol-β-D-glucoside as major anti-biofilm compounds. Interestingly, both compounds disrupt *P. aeruginosa* PAO1 biofilm formation and maintenance with IC_50_ of 1383 µM and 131 µM, respectively. More interestingly, both compounds displayed a synergistic effect with tobramycin with a two-fold increase in its effectiveness in killing biofilm-encapsulated *P. aeruginosa* PAO1. The present study validates the traditional uses of this edible medicinal plant, indicating the therapeutic effectiveness of guava fruits plausibly through the presence of these tri- and tetraterpenoids, which deserve to be tested against pathogens generally implicated in diarrhea.

## 1. Introduction

In the relentless fight against bacterial infections and in front of antibiotic bacterial resistance challenges, the search for new active compounds that could strengthen the therapeutic arsenal against persistent bacterial infections is one of the primary concerns of WHO [1]. Infections by bacteria are mainly related to their ability (i) to invade their hosts and disseminate by means of different types of motility and the release of a myriad of virulence factors which lead to the host cell and tissue damage, and (ii) to grow in “biofilms” that allow both evasion from immune defense systems and resistance to antibiotics. Biofilms are surface-associated bacterial communities enclosed within an extracellular polymeric substances (EPS) matrix, a gel structure composed of macromolecules, mainly exopolysaccharides, proteins, nucleic acids, and lipids [2]. This bacterial lifestyle requires both a modulation of motilities, notably the “*swimming motility*” silencing and “*swarming motility*” activation, which allows effective attachment to the surface, and the production of extracellular matrix components to maintain a solid, protective, and structured community [3]. The protective role of biofilm is a major cause of bacterial persistence, particularly in chronic infections [4], so it is strongly suggested that interfering with biofilm formation and maintenance could lead to improvement in antibiotic effectiveness and accelerate bacterial clearance in chronic infections [5].

For the WHO, *Pseudomonas aeruginosa* represents one of the “priority pathogens” for research and development of new antimicrobials [1] as this opportunistic bacterium presents broad inherent antibiotic resistance [6] and protective biofilms building [7]. Indeed, this bacterium is a frequent cause or aggravating factor of chronic lung and urinary tract infections associated with ineffective antibiotic treatments [8], enterocolitis in children [9], and antibiotic-associated diarrhea in immunocompromised adults [10]. The regulation of biofilm formation by *P. aeruginosa* involves different bacterial machineries, including the quorum sensing (QS), the two-component regulatory system GacS/GacA, the cAMP-Vfr complex, and the second messenger c-di-GMP pool concentration. QS is a cell-to-cell signaling mechanism that induces the expression of various genes related to biofilm development, motilities, and bacterial virulence, including the common virulence factors pyocyanin and rhamnolipids [5]. The QS mechanism is intimately intricated with the two-component regulatory system GacS/GacA which is considered a QS super-regulator [11]. Likewise, the complex formed by the second messenger cAMP and the transcription factor Vfr acts as a QS super-regulator but can also directly inhibit flagella synthesis, meaning the silencing of swimming ability, and triggers the expression of type IV pili (TFP) genes, leading to strong attachments to solid surfaces [12]. Finally, c-di-GMP is universally recognized to control the bacterial transition between the planktonic and biofilm lifestyles, with high c-di-GMP levels activating exopolysaccharide biosynthesis and low c-di-GMP levels enhancing flagella formation and planktonic growth [13].

For thousands of years and long before the massive use of antibiotics, traditional medicines have applied various parts of medicinal plants (roots, bark, leaves, fruits…) to treat chronic and acute infectious diseases. Many of them have shown bactericidal activities against pathogenic bacteria, allowing the isolation of a wealth of original compounds [14]. Within the past two decades, natural compounds from various sources have also been reported to inhibit *P. aeruginosa* biofilm formation and to disrupt its maintenance without affecting bacterial viability. Particularly, medicinal plants used to treat infectious diseases and/or their symptoms are cited as major sources of anti-biofilm natural products [15]. For instance, *Dalbergia trichocarpa* Baker., a medicinal Malagasy plant traditionally used to treat laryngitis and diarrhea, synthesizes an oleanolic aldehyde coumarate that inhibits the formation/maintenance of *P. aeruginosa* PAO1 biofilm at 200 μM [16]. Likewise, *Platostoma rotundifolium* (Briq.) A. J. Paton, widely used in traditional Burundian medicine against microbial diseases, produces di- and triterpenoids, cassipourol, β-sitosterol, and α-amyrin, which inhibit biofilm formation at 12.5, 50, and 50 µM, respectively [17]. These results highlight the relevance of investigating antimicrobial and anti-biofilm properties among anti-infectious medicinal plants and argue for their uses in traditional medicine. However, major issues can be encountered with medicinal plants regarding their quality and safety, and a series of reports point to some unpredictable toxicity issues [18] that could weaken their therapeutic interest. This problem could be minimized by considering edible medicinal plants, i.e., “*ambivalent plants*” that combine therapeutic and nutritive properties, and for which regular culinary use, without reported major adverse side effects, indicates a most probable atoxicity.

The edible medicinal plant *Psidium guajava* L. is a worldwide common and popular traditional remedy for gastrointestinal diseases such as diarrhea, dysentery, and indigestion [19]. During the last decade, *P. guajava* leaves have been widely investigated for their antimicrobial potential including bactericidal and/or anti-biofilm activities against various pathogenic bacteria. For instance, the methanol fraction of *P. guajava* leaves from Peru had an antibacterial effect on *Streptococcus gordonii* and *Porphyromonas gingivalis*, and, at the relatively high concentration of 1.56 mg/mL, decreased biofilm adhesion [20]. Similarly, Dutta et al. [21] demonstrated anti-biofilm activities of leaves methanol extracts, from which benzyl isocyanate, at the concentration of 440 µg/mL, appeared as a major anti-biofilm compound against *Staphylococcus aureus* clinical strains. More interestingly, quercetin and quercetin 3-0-arabinose (at the concentrations of 50 and 100 µg/mL, respectively), obtained from the flavonoid-rich fraction of *P. guajava* leaves, inhibited in *P. aeruginosa* PAO1, pyocyanin, proteolytic and elastolytic enzymes, swarming motility, and biofilm [22]. These studies validate the traditional uses of guava leaves and highlight their importance as a potential source of various anti-biofilm compounds. Interestingly, other parts of guava are also used in traditional medicine. Notably, the edible part, the fruit, is also used during diarrhea treatment and thus could be suggested as a potential source of non-toxic anti-biofilm compounds [23]. In this hypothesis, the present study investigates the antimicrobial properties of guava fruit extracts against *P. aeruginosa* PAO1, including bactericidal, anti-virulence, and anti-biofilm properties, in the perspective of highlighting the presence of relevant original compounds.

## 2. Results

### 2.1. Guava Fruit Extracts Exhibit Weak Bactericidal Activities against P. aeruginosa PAO1

Pulp of ripe guava fruit powders sequential n-hexane (HEX), dichloromethane (DCM) and ethanol (EtOH) dry extracts showed weak bactericidal activities towards *P. aeruginosa* PAO1 with minimum inhibitory concentration (MIC) of 1000 µg/mL, a very high value compared to the positive control tobramycin (MIC, 0.5 µg/mL), an antibiotic generally used to treat patients with cystic fibrosis infected by *P. aeruginosa* [24].

### 2.2. The HEX, DCM, and EtOH Extracts Do Not Affect P. aeruginosa PAO1 Growth

Since the three extracts exhibit very limited bactericidal activities, they were tested for other antibacterial activities targeting important processes for bacterial invasion such as virulence factors production, motilities, and biofilm formation. As a prerequisite, we first verified their impact on *P. aeruginosa* PAO1 growth kinetic by turbidity measurement in comparison with DMSO condition (1%). Sub-MIC concentrations of extracts (100 µg/mL) were selected to ensure that extract absorbance would not interfere with bacterial growth absorbance measurement. Interestingly, the cell growth of PAO1 is not affected, suggesting that bacterial development is not impaired by these extract concentrations at any stage of growth (Figure S1). Thus, this concentration was used for all further experiments.

### 2.3. The HEX and DCM but Not the EtOH Extracts Reduce the Biofilm Formation in P. aeruginosa PAO1

**Figure 1:**
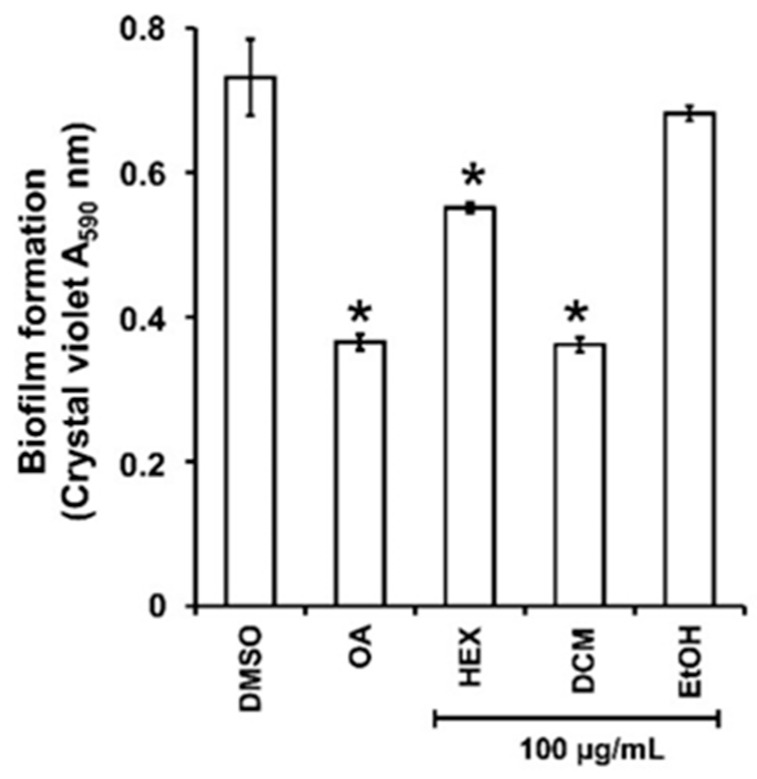
Biofilm formed by *P. aeruginosa* in the presence of guava fruit extracts. The biofilm formation of *P. aeruginosa* PAO1 grown in biofilm broth medium supplemented with DMSO 1% (negative control), oleanolic acid 800 µM (OA, positive control), or hexane (HEX), dichloromethane (DCM), and ethanol (EtOH) extracts at 100 µg/mL after static incubation at 37 °C for 24 h. The biofilm formation was quantified as a measurement of A_590nm_ by crystal violet staining. The experiments were conducted in quintuplicate with three independent assays. Error bars represent the SD; asterisks indicate samples that are significantly different from the DMSO (*p* ˂ 0.05).

As the persistence of bacterial invasion is mainly related to the ability to form protective biofilms [25], the impact of the three extracts on *P. aeruginosa* PAO1 biofilm formation was first evaluated. There was a significant decrease in biofilm biomass when PAO1 wild-type was grown for 24h in the presence of HEX and DCM extracts at 100 µg/mL (Figure 1) with 28 ± 3 and 59 ± 2% inhibition, respectively. By contrast, the EtOH extract did not reduce biofilm formation. Interestingly, the DCM extract impacts biofilm formation as much as the positive control, oleanolic acid (OA) at 800 µM (≈365.36 µg/mL; 56 ± 4% inhibition), and was therefore selected for further investigation.

### 2.4. Bioguided Fractionation of the DCM Extract

**Figure 2:**
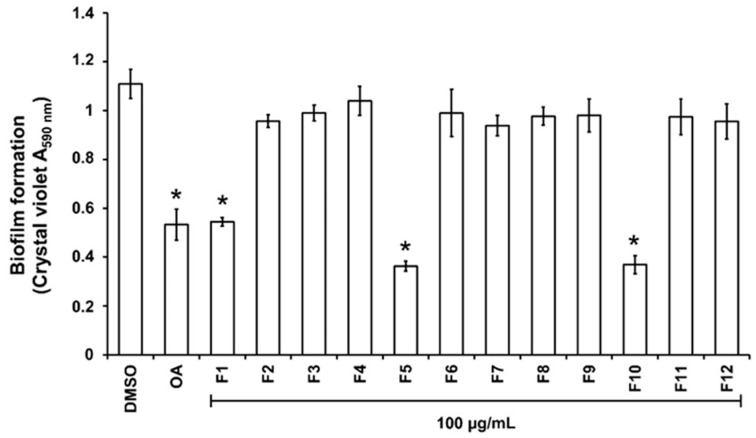
Effect of the fractions obtained from the DCM extract on PAO1 biofilm formation. The biofilm formation of *P. aeruginosa* PAO1 grown in biofilm broth medium supplemented with DMSO 1% (negative control), oleanolic acid 800 µM (OA, as positive control), or dichloromethane (DCM) fractions F1 to F12 at 100 µg/mL after static incubation at 37 °C for 24 h. The biofilm formation was quantified as a measurement of A_590nm_ by crystal violet staining. The experiments were conducted in quintuplicate with three independent assays. Error bars represent the SD; asterisks indicate samples that are significantly different from the DMSO (*p* ˂ 0.05).

**Figure 3:**
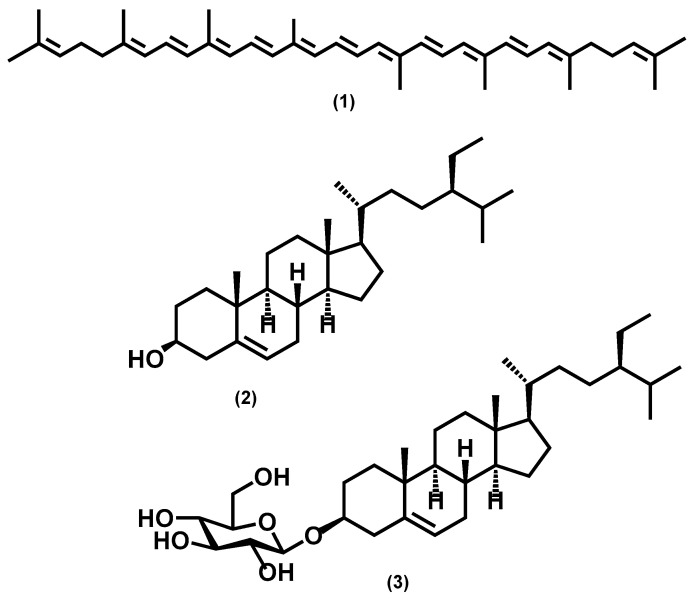
Chemical structures of isolated compounds from *P. guajava*: lycopene (1), β-sitosterol (2), and β-sitosterol-β-D-glucoside (3).

For the purpose of investigating phytochemical groups potentially responsible for anti-biofilm activities, a column chromatography bioguided fractionation of the DCM extract yielded 12 fractions from which 3 (F1, F5, and F10) present a significant inhibitory effect (inhibition % at 100 µg/mL, 50 ± 8, 67 ± 3 and 66 ± 7, respectively) on biofilm biomass (Figure 2). These active fractions were subjected to successive preparative thin-layer chromatographies, allowing the isolation of three major compounds (Figure S2). HPLC-UV-MS, MALDI-TOF-MS, and NMR spectroscopy analysis, detailed in Text S1, Tables S1 and S2, and Figure S3, led to the identification of lycopene (3 mg, from F1 fraction), β-sitosterol (7 mg, from F5) and β-Sitosterol-β-D-glucoside (4 mg, from F10) (Figure 3). β-sitosterol has already been reported for its anti-biofilm activity in our previous study [17], whereas neither lycopene nor β-sitosterol-β-D-glucoside have not been reported yet for such property and were thus further studied.

### 2.5. Lycopene and β-Sitosterol-β-D-Glucoside Reduce PAO1 Biofilm Formation in a Dose-Dependent Manner

**Figure 4:**
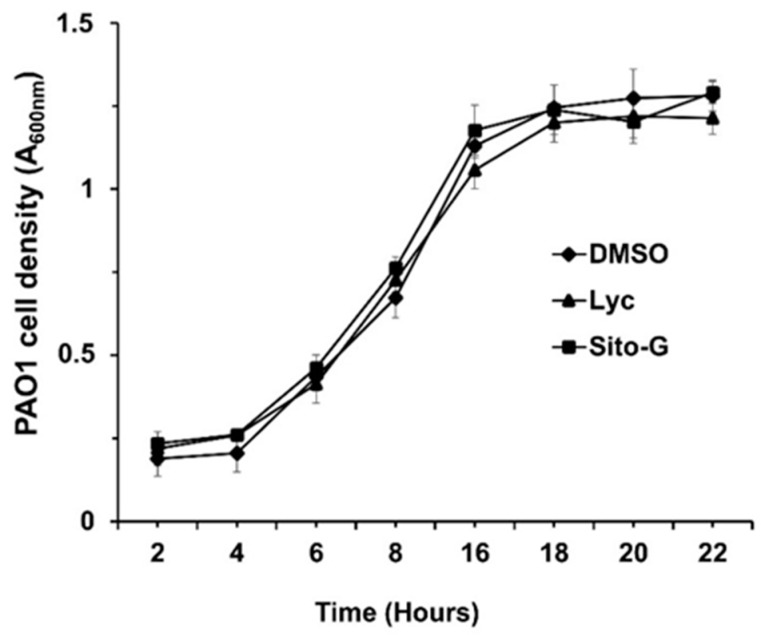
Effect of lycopene (Lyc) and β-sitosterol-β-D-glucoside (Sito-G) on the growth kinetics of PAO1. Growth kinetics of PAO1 in presence of DMSO 1% (negative control) or Lyc (at 1386 µM) or Sito-G (at 131 µM) over a period of 22 h under agitation by measuring the cell density of the bacterial growth at A_600 nm_. The bars represent the standard deviation of the mean. There were no statistical differences between the 3 conditions at any time point (n = 4) (*p* < 0.05).

The anti-biofilm activities of lycopene (Lyc) and β-sitosterol-β-D-glucoside (Sito-G) were tested at different concentrations on PAO1 wild-type grown for 24 h in the presence of each compound. The anti-biofilm activity appears dose-dependent for Lyc with an IC_50_ estimated at 1386 µM (Figure S4). Sito-G inhibits biofilm formation at a concentration down to 21.6 µM with an IC_50_ estimated at 131 µM. To ensure that both compounds, at their anti-biofilm IC_50s_, would not affect the bacterial viability of *P. aeruginosa* PAO1, we evaluated their effects on the kinetic growth of PAO1. As shown in Figure 4, the cell growth kinetics of *P. aeruginosa* PAO1 were neither affected by Lyc nor by Sito-G anti-biofilm IC_50s_ comparatively to the DMSO condition (1%) suggesting that bacterial development is not impaired at any stage of growth. These IC_50s_ were then used to investigate the impact of both compounds on *P. aeruginosa* PAO1 virulence properties including biofilm phenotype and its maintenance, virulence factor production, and motilities.

### 2.6. The DCM Extract, Lyc and Sito-G Affect the Biofilm Phenotype of P. aeruginosa PAO1

**Figure 5:**
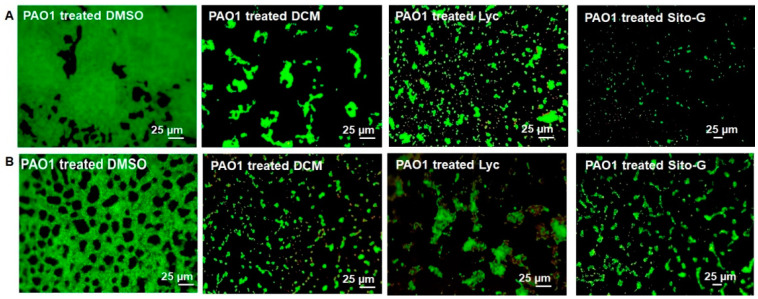
Impact of the dichloromethane (DCM) extract, Lycopene (Lyc), and β-sitosterol-β-D-glucoside (Sito-G) on PAO1 biofilm phenotypes. (**A**) Effect of the DCM extract or Lyc or Sito-G on biofilm development [PAO1 cells were incubated statically at 37 °C for 24 h in presence of DMSO 1% (negative control), or DCM at 100 µg/mL or Lyc at 1386 µM or Sito-G at 131 µM]. (**B**) Effect of DCM extract or Lyc or Sito-G on 24 h preformed biofilm (PAO1 cells were incubated for 24 h and then treated for 24 h with DCM at 100 µg/mL or DMSO 1%, or Lyc at 1386 µM or Sito-G at 131 µM). Cells were visualized after staining with SYTO-9 (LIVE/DEAD BacLight kit) in which green fluorescence color represents living cells. Fluorescence images were captured using an epifluorescence microscope (40× objective lens) equipped with Sony L2 camera.

Considering the important reduction in biofilm biomass in the presence of DCM extract, or Lyc or Sito-G, their impacts on PAO1 biofilm phenotype were explored. As shown in Figure 5, epifluorescence microscopy indicates that the biofilm architecture of *P. aeruginosa* PAO1, grown in static for 24 h with DCM extract at the concentration of 100 µg/mL, is disrupted compared to the control condition (DMSO, 1%). Indeed, the biofilm formed in the presence of DMSO is composed of thick and homogenous layers on coverslips with good and regular cell-to-cell connections interspaced by uncolonized surface (Figure 5A; PAO1 treated DMSO) whereas, in the presence of DCM extract (Figure 5A; PAO1 treated DCM), *P. aeruginosa* PAO1 fails to establish compact cell-to-cell attachment, resulting in a reduction in microcolonies confluence and an increase in uncolonized surface; this observation suggests a lack of biofilm biomass, in coherence with the crystal violet staining measurements (Figure 1). On a 24 h preformed *P. aeruginosa* PAO1 biofilm, the DCM extract leads to a degradation of the compact and heterogeneous biofilm layer (Figure 5B; PAO1 DCM-treated vs. DMSO control), leaving a structure mainly composed of isolated bacterial clumps. These results suggest that DCM extract induces bacterial dispersion out of a pre-formed biofilm.

Similarly to the DCM extract, Lyc and Sito-G affect the biofilm architecture of *P. aeruginosa* PAO1 when added (i) during static growth over 24 h (Figure 5A; PAO1 treated Lyc and PAO1 treated Sito-G); or (ii) on 24 h preformed biofilm experiments (Figure 5B; PAO1 treated Lyc and PAO1 treated Sito-G), with very few biofilm layers being observed in microscopy in both cases.

### 2.7. DCM Extract, Lyc, and Sito-G Increase the Effectiveness of Tobramycin against Biofilm-Encapsulated PAO1

**Figure 6:**
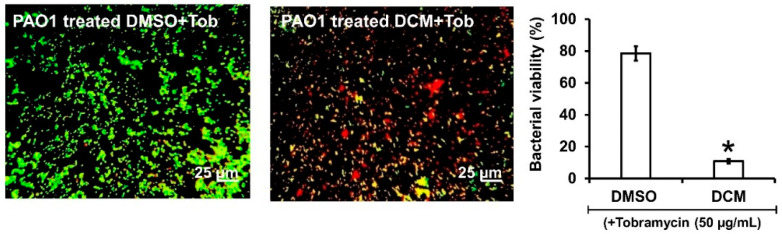
Synergistic activity of DCM with tobramycin against biofilm-encapsulated PAO1. PAO1 cells were incubated statically for 24 h and then treated for 24 h with tobramycin (Tob, 50 µg/mL final concentration) and DMSO 1% (negative control) or DCM (100 µg/mL final concentration). Cells were visualized after staining with SYTO-9 and IPP (LIVE/DEAD BacLight kit) in which green color represents living cells and red color indicates dead cells whereas yellow color indicates the beginning of membrane integrity loss, i.e., probably dying cells. Error bars represent the standard deviation of the mean; The experiments were conducted in quintuplicate with three independent assays, and asterisk indicates significant difference from the DMSO (*p* < 0.05).

As the DCM extract, Lyc and Sito-G negatively impact preformed biofilms, we hypothesized that biofilm-encapsulated bacteria would be more accessible to antibiotic treatments in their presence. Tobramycin was selected as it is widely used to treat acute *P. aeruginosa* exacerbations in patients with cystic fibrosis [26] but appears less effective on biofilm-encapsulated *P. aeruginosa* compared to their planktonic counterparts [27]. Accordingly, the effectiveness of tobramycin (50 μg/mL) combined with DCM extract (100 μg/mL) was evaluated on 24 h preformed biofilms. Interestingly, the addition of the DCM extract considerably improves the effectiveness of tobramycin (50 µg/mL) against PAO1 (Figure 6), a drastic proportion (89 ± 3%) of biofilm-encapsulated bacteria being killed vs. 22 ± 4% with tobramycin alone.

**Figure 7:**
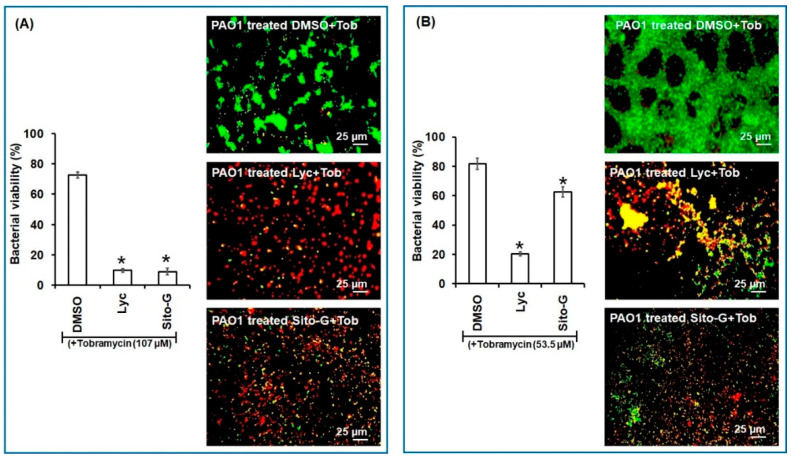
Synergistic activity of lycopene (Lyc) and β-sitosterol-β-D-glucoside (Sito-G) with tobramycin against biofilm-encapsulated PAO1. (**A**) PAO1 cells were incubated statically for 24 h and then treated for 24 h with tobramycin (Tob) at 107 µM final concentration and DMSO 1% (negative control) or Lyc (1386 µM final concentration) or Sito-G (131 µM final concentration). (**B**) PAO1 cells were incubated statically for 24 h and then treated for 24 h with tobramycin at 53.5 µM final concentration and DMSO 1% or Lyc (1386 µM final concentration) or Sito-G (131 µM final concentration). Assessment of bacterial viability and microscopy were performed as in Figure 6. Red color indicates dead cells and green color indicates living cells whereas yellow colors indicate the beginning of membrane integrity loss and then considered as dying cells. Error bars represent the standard deviation of the mean; all experiments were performed in quintuplicate with three independent assays, and asterisk indicates significant difference from the DMSO (*p* < 0.05).

Similarly, as shown in Figure 7, both Lyc and Sito-G significantly increase the effectiveness of tobramycin. Indeed, when tobramycin (107 µM, 50-fold above its planktonic MIC) was combined with Lyc (1386 µM) or sito-G (131 µM) and added to one-day-old biofilm cultures, the proportion of dead cells was 90 ± 3% compared to 27 ± 4% for the control tobramycin-DMSO. These results suggest an improvement of antibiotic diffusion/penetration through the disrupted biofilm matrix and corroborate the observed synergizing effects of the DCM extract. Interestingly, even when reducing by two-fold the tobramycin concentration (53.5 µM), the proportion of dead cells in the presence of Lyc or Sito-G is still significantly higher (79 ± 6% and 37 ± 4%, respectively, versus 18 ± 3% for DMSO + tobramycin).

### 2.8. The Three Organic Extracts, Lyc and Sito-G Have No Influence on the Production P. aeruginosa PAO1 Pyocyanin and Rhamnolipids as Well as on the Expression of QS-Dependent rhlA and lasB Genes

In the purpose of exploring other anti-virulence activities and considering that the severity of infection by *P. aeruginosa* is mainly related to its ability to release a myriad of virulence factors, including pyocyanin and rhamnolipids [5], we evaluated the impact of the three extracts (HEX, DCM, and EtOH) and isolated compounds from the DCM extract on the production of *P. aeruginosa* PAO1 pyocyanin and rhamnolipids as well as on the expression of QS-dependent *rhlA* and *lasB* genes, encoding for 3-(3-hydroxyalkanoyloxy) alkanoic acids (precursor of rhamnolipids) and elastase B, respectively. As shown in Figure S5, the three organic extracts, Lyc and Syto-G do not significantly decrease the production of pyocyanin (6 ± 2%, 7 ± 5%, 7 ± 2%, 12 ± 5%, and 11 ± 2% of inhibition, respectively), and rhamnolipids (6 ± 3%; 8 ± 5%, 3 ± 2%, 8 ± 5% and 13 ± 5% of inhibition, respectively), by *P. aeruginosa* when compared to the DMSO control while the positive control naringenin inhibits both virulence factors production (45 ± 5% and 48 ± 2% of inhibition, respectively). Similarly, the three organic extracts nor Lyc and Sito-G do not negatively impact *lasB* and *rhlA* gene expression suggesting that they neither disrupt the QS circuitry.

### 2.9. DCM Extract and Lyc but Not HEX, EtOH and Sito-G Affect P. aeruginosa PAO1 Swarming Motilities

**Figure 8:**
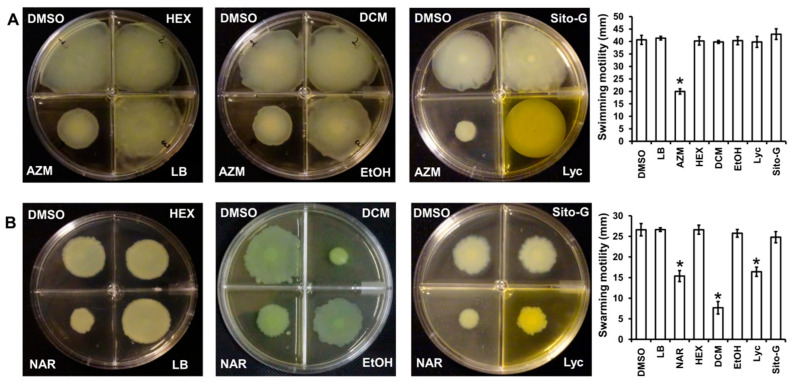
Effect of the three organic extracts, lycopene (Lyc) and β-sitosterol-β-D-glucoside (Sito-G) on *P. aeruginosa* PAO1 motilities (**A**) swimming ability of PAO1 onto LB agar (0.3%) supplemented with DMSO (1%, negative control), hexane (HEX, 100 µg/mL final concentration), dichloromethane (DCM, 100 µg/mL final concentration) or ethanol (EtOH, 100 µg/mL final concentration) extracts, Lyc (1386 µM final concentration) or Sito-G (131 µM final concentration) or azithromycin (AZM, 2.6 µM final concentration). (**B**) Swarming motility of PAO1 onto LB agar (0.6%) supplemented with glutamate (0.05%) and glucose (0.2%) and DMSO (1%), HEX (100 µg/mL final concentration), DCM (100 µg/mL final concentration) or EtOH (100 µg/mL final concentration) extracts, Lyc (1386 µM final concentration) or Sito-G (131 µM final concentration) or naringenin (NAR, 1000 µM final concentration). After incubation at 37 °C for 24 h, the migration zones from the point of inoculation were measured for each condition and expressed in mm. Error bars represent the standard deviation of the mean; the experiments were conducted in quintuplicate with three independent assays and asterisks indicate samples that are significantly different from the DMSO (one-way ANOVA followed by Dunnett’s test (*p* ˂ 0.05).

As the three organic extracts, Lyc and Sito-G do not affect the ability of *P. aeruginosa* to produce virulence factors, we investigated their impact on bacterial dissemination through motilities, factors strongly associated with *P. aeruginosa* pathogenesis through migration and attachment to surfaces [28]. Two types of motilities were evaluated, the swimming motility that needs active flagella and the swarming motility that requires both flagella and TFP [29]. As shown in Figure 8A, none of the three extracts at 100 µg/mL reduces the swimming motility, contrary to the positive control azithromycin at 2.6 µM [30], whereas only the DCM extract significantly decreases the swarming motility (82 ± 3% inhibition), two-fold higher compared to the positive control naringenin (40 ± 3% inhibition at 1000 µM) (Figure 8B). Interestingly, both isolated compounds from DCM extract do not negatively impact swimming motility, whereas only Lyc significantly decreases swarming motility (52 ± 3% inhibition) similar to the positive control naringenin (40 ± 3% inhibition) (Figure 8B). As we previously showed that β-sitosterol does not negatively impact swarming motility [17], the observed anti-swarming of the DCM extract is probably linked to the presence of Lyc.

## 3. Discussion

*P. guajava* L. is a widely cultivated fruit plant, distributed worldwide in tropical and subtropical areas. Guava fruit is rich in vitamins A and C, iron, phosphorus, calcium, and minerals and contains various organic compounds such as (i) polyphenols with potential antimicrobial activities [31], notably the gallic and ferulic acids active against a methicillin-resistant *S. aureus* clinical strain [32] and (ii) sesquiterpenes with depressant activities on the central nervous system [33].

To the best of our knowledge, this is the first study highlighting the presence of lycopene, a carotenoid that perturbs *P. aeruginosa* biofilm formation and maintenance, allowing to increase the effectiveness of tobramycin against biofilm-encapsulated bacteria. Likewise, two other triterpenoids, β-sitosterol and β-sitosterol-β-D-glucoside, isolated from the same dichloromethane extract, exhibit similar anti-biofilm activity against *P. aeruginosa* PAO1. The presence of these three terpenoids, which are considered atoxic [34,35], probably justifies the claimed therapeutic effectiveness of guava fruits.

Recently, we have shown anti-biofilm activities for other carotenoids, particularly the xanthophyll lutein [5] that inhibits the *P. aeruginosa* PAO1 biofilm formation and maintenance at the concentration of 22 µM. Intriguingly, the effective concentration of lycopene is 60-fold higher compared to lutein (1386 µM versus 22 µM) suggesting a probable importance of hydroxylated β- and/or ε-ionone rings in the lutein skeleton. This hypothesis needs deeper structure–activity relationship investigation by exploring the anti-biofilm activities of various representative compounds in the carotenoids family.

Considering the main key actors involved in biofilm regulation, the mechanism of action of lycopene and lutein in disrupting biofilm formation and maintenance seems different as lutein impacts both the QS and Vfr pathways [5] whereas lycopene has no influence on *P. aeruginosa* QS circuitry, including *lasB* and *rhlA* genes, and on QS-regulated virulence factor production (pyocyanin and rhamnolipids) (Figure S5). Accordingly, a deeper investigation of plausible lycopene mechanisms of action should be initiated toward other biofilm key processes such as c-di-GMP pool regulation, exopolysaccharide biosynthesis, and TFP gene expression [36]. Indeed, the observed inhibition of swarming motility (Figure 8) could be linked to a reduction in TFP gene expression, leading to failure in bacteria attachment to surfaces. Likewise, the observed biofilm degradation and the plausible loss of its protective ability (Figure 5, Figure 6 and Figure 7) could be linked to a massive reduction in matrix synthesis, particularly exopolysaccharides, that could result from a disruption of c-di-GMP production [37] or from its hydrolysis. Consequently, this disrupted biofilm matrix could explain the synergism of Lyc and Sito-G with tobramycin through an improvement of antibiotic diffusion/penetration as it has already been demonstrated that the extracellular matrix protects *P. aeruginosa* biofilms by limiting the penetration of tobramycin [38].

β-Sitosterol and its β-D-glucoside present close active concentrations, with IC_50_ at 200 and 131 µM, respectively [17], but do not exhibit the same spectrum of activity. Indeed, β-sitosterol has been shown to inhibit the production of pyocyanin and rhamnolipids contrary to Sito-G, suggesting a loss of activity following glycosylation and an unlikely in situ deglycosylation.

The therapeutic use of edible medicinal plants may have the distinct advantage of combining anti-virulence and/or anti-biofilm properties with a nutritional apport of additional energy, vitamins, and minerals that support immune cells, both in their role and in their resistance to bacterial attacks [39]. Indeed, pathogenic bacteria such as (i) *Vibrio cholera*, responsible for severe diarrhea, can form biofilms on human leukocytes, leading to their death; and (ii) *P. aeruginosa* attack and eliminate the attracted leukocytes with their virulence factors, notably rhamnolipids [40]. More interestingly, the ability of guava fruit to reduce the effective concentrations of antibiotics required could reduce the risk of diarrhea due to flora imbalances linked with massive antibiotherapy. Indeed, long courses of antibiotic therapy likely increase infection susceptibility via a loss of normal fecal microbiota, leading to reduced competitive exclusion and colonic barrier protection [41].

In conclusion, the relevance of guava fruit consumption during diarrhea relies on proven antibacterial, antioxidant, and anti-biofilm activities, indicating a particular and affordable therapeutic interest for low-income countries; In addition to previously reported compounds (phenolic acids, sesquiterpenoids), the presence of three anti-biofilm terpenoids suggests the presence of a diversified array of anti-infectious compounds in the entire fruit, a true defense cocktail aimed at various microbial targets with high therapeutic relevance. From this perspective, it appears important to investigate the antibacterial effects of these compounds against other relevant pathogens largely implicated in diarrhea such as *Escherichia coli* and *Salmonella.*

## 4. Material and Methods

### 4.1. Bacterial Strains and Growth Condition

*P. aeruginosa* wild-type and PAO1 reporter strains were grown (37 °C, agitation 175 rpm) in LB-MOPS broth, purchased from Sigma-Aldrich (50 mM, pH 7) and supplemented with carbenicillin (300 μg/mL) when appropriate. Plasmids (pβO1 and pβO2 which are pQF50-derivatives containing P*_lasB_*-lacZ and P*_rhlA_*-lacZ transcriptional fusion, respectively), were used and introduced in PAO1 as previously described [42]. PAO1 was provided by Laboratoire de Biotechnologie Végétale (LBV, Université Libre de Bruxelles), Brussel, Belgium.

### 4.2. Chemicals and Solvents

Antimicrobial drugs (carbenicillin, tobramycin, and azithromycin) were purchased from TCI^®^ (Tokyo chemical industry Co., Ltd., Tokyo, Japan) and dissolved in deionized water. Naringenin and oleanolic acid were purchased from Sigma-Aldrich and dissolved in 100% DMSO. Analytical grade solvents were obtained from VWR International (Leuven, Belgium) and redistilled before use. All other chemicals were also analytical grade and purchased from Sigma Aldrich (St. Louis, MO, USA).

### 4.3. Plant Material and Extraction

*P. guajava* L. fruits were collected from the Analamanga area in Antananarivo rural province (Madagascar). Plant extraction was conducted as previously described, with the purpose of generating residue for antibacterial screening tests [17]. Briefly, the dried and powdered *P. guajava* L. fruits (5 g) were successively extracted with 3 solvents of increasing polarities (HEX, DCM, and EtOH), 50 mL each for 24 h maceration at room temperature with continuous agitation at 250 rpm. The extracts were filtered through Whatman No.1 filter paper, dried in a rotary evaporator, under vacuum, and stored at 20 °C until use. Each extraction yielded 12 mg, 23 mg, and 79 mg of residues, respectively, and a portion of each extract was dissolved in dimethylsulfoxide (DMSO) to obtain concentrations appropriate for biological tests. A second extraction protocol was conducted with the purpose of generating enough residues for fractionation and isolation of active compounds. Thus, dried powdered samples of *P. guajava* fruits (100 g) were statically macerated overnight at room temperature in 1 L of dichloromethane and percolated with 2 L dichloromethane (1 L/h). The collected dichloromethane extracts were filtered on Whatman paper, and evaporated under vacuum; the resulting residue (567 mg) was stored at −20 °C.

### 4.4. Chromatographic Purification of Major Bioactive Compounds from P. guajava *L.* DCM Extract

For chromatographic purification, the DCM extracts were dissolved in 10 mL of n-hexane, loaded onto a chromatography column (35 × 4 cm i.d.) filled with silica gel 60 F254 (63–200 μm/70–230 mesh; Merck) and eluted with DCM with a step gradient of methanol (99:1 to 10:100) to afford 12 fractions (fractions F1 to F12). The fractions were evaporated and stored at −20 °C. The active fractions (F1, F5, and F10) with the best biofilm inhibitory effects were further fractionated and purified by preparative TLC using silica gel RF254 Merck; mobile phases, HEX–toluene (95:5, *v*/*v*) for F1; DCM–methanol (96:4, *v*/*v*) then HEX–acetone (30:70, *v*/*v*) for F5; DCM–methanol-toluene (45:5:5, *v*/*v*) then –methanol (85:15, *v*/*v*) for F10; detection under UV light (254 and 366 nm) and visible light after treatment with 10% (*v*/*v*) H_2_SO_4_ in 95% EtOH followed by heating at 110 °C for 10 min.

### 4.5. Identification and Structure Elucidation of Major Bioactive Compounds by HPLC-UV-MS, MALDI-TOF-MS and NMR

For the purpose of identification and structure elucidation, the purified compound from F1 was analyzed on HPLC-UV-MS by injecting 10 µL (1 mg/mL) in an HPLC-UV-MS system (Agilent 1260 Infinity II system, coupled to Advion Expression CMS L); the column was an RP C18 (Luna^®^ 5 µm C18(2) 100 Å, 250 × 4.6 mm i.d.) with a mobile phase acetonitrile–methanol (50:50, *v*/*v*), under isocratic conditions, at a flow rate of 1 mL/min. The MS LockSpray ion source was operated in positive electrospray ionization (ESI) mode under the following specific conditions: capillary voltage, 120 V; source temperature, 250 °C; desolvation gas temperature, 200 °C; desolvation gas flow, 550 L/h; mass range, 250 to 1000 Da. Nitrogen (>99.5%) was employed as desolvation and cone gas. The MS data analysis was performed using Advion Data Express software 6.6.35.2 referring to the literature data.

Chemical structures of purified compounds from fractions F5 and F10 were elucidated through NMR experiments. Both compounds were dissolved in deuterated chloroform (CDCl3) and 1H and 13C spectra were collected on 500 MHz and 125 MHz NMR instrument BRUKER, model AVANCE III, 14.1. and 9.4 Tesla, respectively. Tetramethyl silane was added as reference. Topspin 1.3 software (Bruker) was used to transform and integrate the NMR signals. The exact masses of purified compounds were confirmed by MALDI-TOF-MS, as previously described by Chapagain and Wiesman [43]. Briefly, isolated compounds were dissolved in chloroform–methanol (85:15, *v*/*v*) to about 1 mg/mL and mixed 1–2 with a 20 mg/mL solution of 2,5-dihydroxybenzoic acid matrix in 90% methanol. For MALDI analyses, 1 µL of the mixture was directly applied to a stainless steel MALDI target, allowed to dry, and analyzed in a Reflex IV (Bruker Daltonik, Bremen, Germany) MALDI-TOF mass spectrometer with nitrogen laser irradiation at 337 nm; positive ion mode; accelerating voltage, 20 kV; reflectron mode; mass range, 450 to 2400 *m*/*z*.

To confirm the structure of isolated compounds, collected data were compared with references data from literature [44,45,46,47,48,49].

### 4.6. Determination of Minimum Inhibitory Concentration and Assessment of Kinetic Bacterial Growth

For MIC (minimum inhibitory concentration) and MBC (minimum bactericidal concentration) determination, *P. aeruginosa* PAO1 was grown on 24-well microplates with 1 mL of LB broth, in presence of *P. guajava* L. fruit extracts or fractions or purified compounds in different concentrations (from 31.25 to 2000 µg/mL), at 37 °C for 24 h. The MIC was defined as the lowest antimicrobial concentration that completely inhibited growth as detected by the naked eye. The culture of the strains is completed by the microdilution method on a 96-well microplate as previously described [50]. All inhibited growth cultures were then sub-cultured onto LB agar plate and incubated at 37 °C for 24 h to determine the MBC, which was defined as the lowest concentration that yielded negative sub-cultures [50]. Tobramycin, an antibiotic widely used to treat *P. aeruginosa* lung infection in cystic fibrosis patients was selected as positive control [29]. Additionally, the effect of *P. guajava* L. fruit extracts or active compounds on *P. aeruginosa* PAO1 growth kinetic was assessed by evaluating PAO1 cell density at A_600nm_ with a SpectraMax M2 device (Molecular Devices, Silicon Valley, CA, USA) over 22 h culture.

### 4.7. Quantitative Analysis of Pyocyanin and Rhamnolipids Production

The production of pyocyanin and rhamnolipids was assessed as previously described [51,52]. *P. aeruginosa* PAO1 was grown for 18 h in liquid LB-MOPS. PAO1 cell suspension (50 µL) was added to 1 mL of LB-MOPS (starting A_600nm_ ranging between 0.02 and 0.025) supplemented with 10 µL of *P. guajava* L. fruit extracts or purified compounds dissolved in DMSO (100 µg/mL) or DMSO (final DMSO concentration, <1%, *v*/*v*). After 18 h, samples were taken to measure growth (at A_600nm_) and pyocyanin (at A_380nm_ after extraction in CHCl_3_ and back-extraction in 0.2 M HCl) as well as rhamnolipids (at A_638nm_ after extraction by a methylene-blue-based method) with a SpectroMax M2 device (Molecular Devices, San Jose, CA, USA).

### 4.8. Biofilm Formation and Quantification

The quantification of biofilm was performed on 24-well microplates by using crystal violet staining protocol, as previously described [52]. Briefly, an overnight culture of *P. aeruginosa* PAO1 was washed twice and diluted in fresh biofilm broth (BB) medium (Na_2_HPO_4_, 1.25 g/L; FeSO_4_·7H_2_O, 0.0005 g/L; glucose, 0.05 g/L; (NH_4_)_2_SO_4_, 0.1 g/L; MgSO_4_·7H_2_O, 0.2 g/L and KH_2_PO_4_, 0.5 g/L). The diluted cultures (50 µL) were added to 940 µL of BB medium (initial A_600nm_ of the culture between 0.14 and 0.16) and supplemented with 10 µL of the desired solution. DMSO (1% final concentration) and OA (800 µM, final concentration) were used as negative and positive controls, respectively [53]. Purified compounds were tested at different concentrations in the ranges 5.8 to 1488 µM for Lyc and 5.4 to 1384 µM for Sito-G. After static incubation for 24 h at 37 °C, the formed biofilm was stained by crystal violet and A_590nm_ was measured with a SpectroMax M2 device (Molecular Devices, San Jose, CA, USA).

### 4.9. Epifluorescence Microscopy Study of Biofilm Phenotypes and of the Synergism between Tobramycin and DCM Extract or Purified Terpenoids

The *P. aeruginosa* PAO1 biofilm was examined in epifluorescence microscopy, as previously described by Mahavy et al. [5]. The effects of Lyc (1386 µM) or Sito-G (131 µM) or DMSO (1%, *v*/*v*) on biofilm development and on 24 h preformed biofilms were evaluated, applying the same culture conditions as described above. After 24 h incubation, the biofilm development was visualized using the LIVE/DEAD^®^ BacLight™ bacterial viability kit (ThermoFisher Scientific, San Jose, CA, USA). The growth medium was removed and replaced by 500 µL of a solution of SYTO 9 and propidium iodide, diluted 400-fold in BB medium. The biofilms were then incubated for 15 min, and *P. aeruginosa* PAO1 cells were observed under an epifluorescence microscope equipped with a Sony L2 camera using a 40× objective lens; the images were color-enhanced and compiled using Microsoft Photos software 2024.11030.22001.0 (Microsoft Corporation).

For the second assay, PAO1 cells were cultivated statically in BB medium for 24 h at 37 °C in 24-well polystyrene plates to establish biofilms. Lyc, Sito-G, or DMSO was added and incubated for a further 24 h, and the biofilm development and bacterial viability in biofilms were similarly assessed.

The bactericidal activities of tobramycin combined with Lyc 1386 µM or Sito-G 131 µM or DMSO 1% in one-day-old biofilm-encapsulated PAO1 cells were also assessed. PAO1 cells were grown statically in BB medium for 24 h at 37 °C in 24-well polystyrene plates to form biofilms. Tested molecules as described above, and tobramycin (50 µg/mL or 25 µg/mL = 107 µM or 53.5 µM) were added and incubated for a further 24 h and the biofilm development and bacterial viability in biofilms were assessed as described for the first assay. The proportion of live or dead bacteria was estimated by counting the observed green and red or yellow cells in five different fields [54].

### 4.10. Motility Assay

Swarming and swimming motilities were examined by using LB agar (0.6 and 0.3%, respectively), as previously described by Rasamiravaka et al. [52]. After sterilizing and cooling (45–50 °C) LB agar, the test solutions were added, i.e., DMSO 1% (negative control), Lyc 1386 µM or Sito-G 131 µM. Additionally, naringenin at 1000 µM [55] and azithromycin at 2.6 µM [27] were used as positive control. The medium was poured into compartmented Petri dishes and allowed to cool at room temperature. Five microliters of bacterial culture (A_600nm_ = 1) were inoculated at the center of each compartment of the Petri dishes and incubated at 37 °C for 24 h. Bacteria spreading from the inoculation spot were measured with sliding caliper.

### 4.11. Gene Expression and β-Galactosidase Measurements

To monitor gene expression of QS-dependent (*lasB* and *rhlA*), the *β*-galactosidase activity induced by reporter genes was measured using o-nitrophenyl-*β*-D-galactopyranoside [51,55]. After growth in liquid LB-MOPS carbenicillin at 37 °C and 175 rpm for 18 h, PAO1 reporter strains were washed twice in fresh LB medium and resuspended in liquid LB-MOPS-Carbenicillin. PAO1 reporter strains inoculums (50 μL) were incubated (37 °C with 175 rpm agitation) for 18 h in 1 mL LB-MOPS carbenicillin (initial A_600nm_ of culture comprised between 0.020 and 0.025) supplemented with 10 μL of Lyc (1386 µM) or Sito-G (131 µM) dissolved in DMSO or 10 μL of DMSO. Additionally, the flavanone naringenin (1000 µM), known as QS quencher [56], was used as positive control. After incubation, the bacterial density was assessed by spectrophotometry (A_600nm_), and the gene expression by the β-galactosidase assay.

### 4.12. Statistics

All experiments were conducted with a minimum of four replicates and repeated in three independent assays. The statistical significance of data (*p*-value < 0.05) was analyzed using Student’s *t*-tests or one-way ANOVA with post hoc Dunnett’s tests via GraphPad Prism 8 software.

## Data Availability

Data are contained within the article and supplementary materials.

## Supplementary Materials

The following supporting information can be downloaded at: https://www.mdpi.com/article/10.3390/plants13081122/s1, Figure S1: Effect of organic Guava fruit extracts on the growth kinetics of PAO1; Figure S2: Scheme of extractions and bioguided fractionation of dichloromethane Guava fruit extract; Figure S3: HPLC-UV-MS analysis of Lycopene; Figure S4: Dose-dependent anti-biofilm activity of Lyc and Sito-G; Figure S5: Anti-virulence effects of organic Guava fruit extracts and isolated compounds in *P. aeruginosa* PAO1; Text S1: Phytochemical characterization of isolated compounds.
