# Supplementary material for: Edible Medicinal Guava Fruit (Psidium guajava L.) Are a Source of Anti-Biofilm Compounds against Pseudomonas aeruginosa
Source: Plants (Basel). 2024 Apr 17;13(8):1122. doi: 10.3390/plants13081122 (PMC11054768; doi:10.3390/plants13081122)
Supplement: Supplementary file 1 [file plants-13-01122-s001.zip › Text S1. Phytochemical characterization- proofread version.pdf]

Text S1. Phytochemical characterization of isolated compounds:

Compound 1 from fraction F1 was analyzed by HPLC-UV-MS. The chromatograms monitored at 448 nm highlight the presence of a major peak at 35.2 min; its UV spectrum is characteristic of a carotenoid with transition peaks at 468 (shoulder) and 444/498 [44-47]. The UV and mass spectra of this major compound match with those of lycopene and/or its isomers (Molecular Weight = 536 that corresponds to molecular formula, $C_{29}H_{50}O$). To support this hypothesis, the analysis of a lycopene commercial reference in the same HPLC conditions showed identical retention time and UV/Mass spectra. Moreover, their co-injection showed no difference in the retention times, suggesting that the active compound 1 is effectively lycopene. (Figure S3).

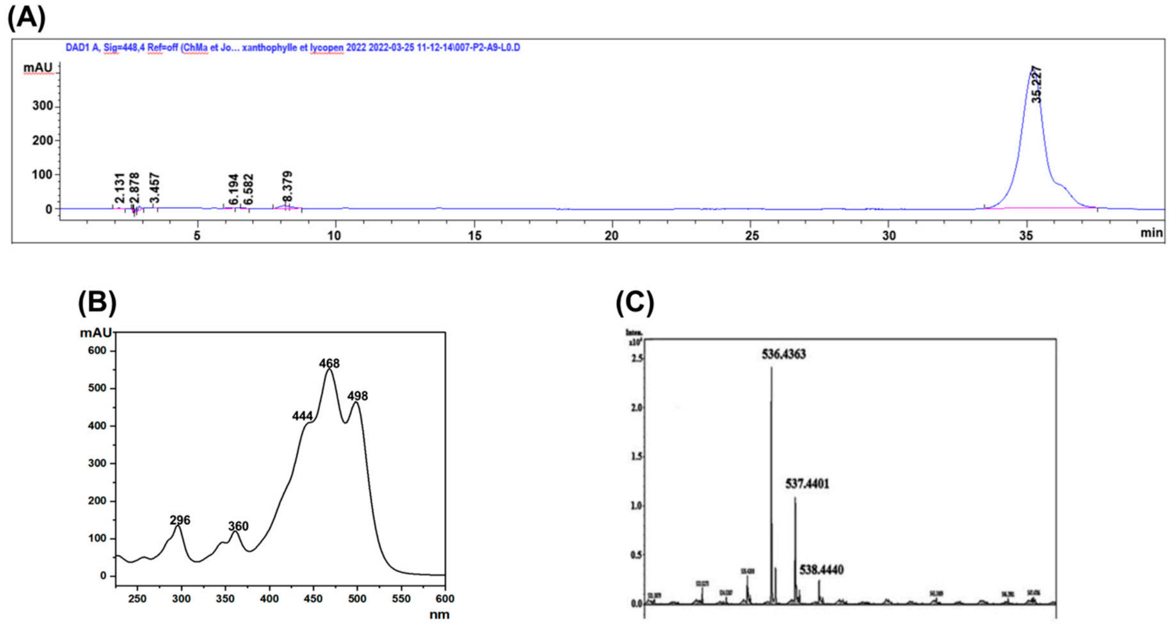

Figure S3 : HPLC-UV-MS analysis of Lycopene

(A) HPLC profile of lycopene monitored at 448 nm; (B) UV-DAD spectra of lycopene; (C) Mass spectra of lycopene isolated from *P. guajava*

Compounds 2 and 3 fraction F5 and F10, respectively were isolated as amorphous white solids and analyzed by NMR and MALDI-TOF.

MALDI-TOF analysis of compound 2 revealed a pseudomolecular ion at m/z 397.381 $[M+H]^+$. The ^{1}H-NMR spectrum ($CDCl_3$, 500 MHz) of this compound (ppm) showed the presence of six methyl signals that appeared as two methyl singlets at δ_H 0.66, and 0.98 which corresponds to the angular methyl singlets. Three methyl doublets appeared at δ_H 0.79, 0.82, and δ_H 0.90, and a methyl triplet at δ_H 0.81 corresponding to the methyl protons. A proton corresponding to the hydroxyl group appeared as a multiplet at δ_H 3.50. The ^{13}C-NMR spectrum ($CDCl_3$, 125 MHz) showed twenty-nine carbon signals made up of six methyl, eleven methylenes, nine methines and three quaternary carbon signals. The ^{13}C-NMR also showed recognizable signals at δ_C 140.1 ppm and δ_C 121.9 ppm which are typical of alkene double bonds. The values at δ_C 19.6 and δ_C 12.1 ppm correspond to angular methyl carbon atoms. The signal at δ_C 72.0 ppm is assignable to the beta hydroxyl group attached to the carbon at position 3. Based on these results (Table S1) and by comparison with literature data, the spectrum peaks could be assigned to β-sitosterol [17].

MALDI-TOF data for compound 3 showed a pseudomolecular ion peak $[M+H]^+$ at m/z 576 suggesting the molecular formula $C_{35}H_{60}O_6$. The ^{1}H-NMR spectra of this compound showed characteristic resonances for β-sitosterol (δ_H 5.4 for the olefinic proton and δ_H 3.79 for the carbonilyc proton). A resonance at δ_H 4.9 is due to an anomeric proton indicating the presence of a glycoside linkage which was confirmed by resonances at δ_H 4.06, 4.08, 4.08, 3.97 and 4.4 which were assigned to H-2′, H-3′, H-4′, H-5′ and H-6′, respectively and the sugar was identified as glucose. The ^{13}C-NMR resolved 35 carbon resonances with characteristic sitosterol olefinic resonances at δ_C 140.5 and δ_C 121.91. HSQC correlation allowed to assign the resonance at δ_C 100.8 to the anomeric carbon. HMBC experiments confirmed the attachment of the sugar at C-3 and the methylene at δ_C 61.65 was assigned to the C-6′ of the glucose.

Table S1 : ^{1}H and ^{13}C NMR spectral data of β-sistosterol

		Present study		Literature [48]	
C	CH_n	δ_C (ppm)	δ_H (ppm)	δ_C (ppm)	δ_H (ppm)
1	CH_2	37.5	1.83	37.21	1.47
2	CH_2	31.9	1.82	31.61	1.57
3	CH	72.0	3.50	71.81	3.51
4	CH_2	42.5	2.27	42.29	2.32
5	C	140.1		141.71	
6	CH	121.9	5.33	121.73	5.34
7	CH_2	32.1	1.97	31.91	2.01
8	CH	32.1	1.42	31.89	1.67
9	CH	50.3	0.91	50.08	1.52
10	C	36.7		36.48	
11	CH_2	21.3	1.47	21.05	1.50
12	CH_2	40.0	1.98	39.73	1.49
13	C	42.5		42.25	
14	CH	57.0	0.97	56.72	1.50
15	CH_2	24.5	1.56	24.28	1.59
16	CH	28.5	1.82	28.23	1.93
17	CH	56.3	1.09	55.99	1.47
18	CH_3	12.1	0.66	11.84	0.66
19	CH_3	19.6	0.98	19.38	1.03
20	CH	36.4	1.34	36.12	1.61
21	CH_3	19.0	0.90	18.75	0.94
22	CH_2	34.2	1.30	33.90	0.93
23	CH_2	26.3	1.14	25.99	1.15
24	CH	46.1	0.91	45.78	1.38
25	CH	29.4	1.64	29.08	1.64
26	CH_3	20.0	0.81	19.81	0.84
27	CH_3	19.4	0.79	18.99	0.62
28	CH_2	23.3	1.25	23.02	1.08
29	CH_3	12.2	0.82	11.96	0.83

Table S2 : ^{1}H and ^{13}C NMR spectral data of Sito-G

		Present study		Literature [49]	
C	CH_n	δ_C (ppm)	δ_H (ppm)	δ_C (ppm)	δ_H (ppm)
1	CH_2	36.92	1.81	36.85	1.25
2	CH_2	29.26	2.12	29.12	1.33
3	CH	76.98	3.79	76.99	3.13
4	CH_2	40	2.69	42.14	2.14
5	C	140.5		140.51	
6	CH	121.91	5.4	121.29	5.34
7	CH_2	31.54	1.98	31.41	1.73
8	CH	31.67	1.44	31.46	1.22
9	CH	49.85	0.96	49.83	1.22
10	C	36.92		36.27	
11	CH_2	20.72	1.5	20.21	1.33
12	CH_2	38.36	2.05	38.20	1.33
13	C	39.42		41.91	
14	CH	56.42	1	56.36	1.22
15	CH_2	23.94	1.61	23.79	1.73
16	CH_2	27.76	1.91	27.76	1.73
17	CH	55.71	1.15	55.66	1.73
18	CH_3	11.54	0.73	11.27	0.62
19	CH_3	19	1.00	19.10	0.94
20	CH	35.80	1.44	35.70	1.32
21	CH_3	18.69	1.04	18.69	0.84
22	CH_2	33.63	1.44	33.51	1.73
23	CH_2	25.71	1.35	25.64	1.73
24	CH	45.52	1.03	45.49	1.12
25	CH	28.89	1.74	28.74	2.14
26	CH_3	18.69	0.93	18.69	0.75
27	CH_3	18.39	0.92	18.35	0.73
28	CH_2	22.71	1.29	22.60	1.33
29	CH_3	11.63	0.96	12.29	0.77
1'	CH	100.85	4.9	100.82	4.11
2'	CH	73.30	4.06	73.21	3.14
3'	CH	76.17	4.08	76.18	3.14
4'	CH	69.97	4.08	69.90	3.14
5'	CH	75.51	3.97	75.62	3.06
6'	CH_2	61.65	4.4	61.36	2.94
