# Supplementary figures and images for: Edible Medicinal Guava Fruit (Psidium guajava L.) Are a Source of Anti-Biofilm Compounds against Pseudomonas aeruginosa
Source: Plants (Basel). 2024 Apr 17;13(8):1122. doi: 10.3390/plants13081122 (PMC11054768; doi:10.3390/plants13081122)

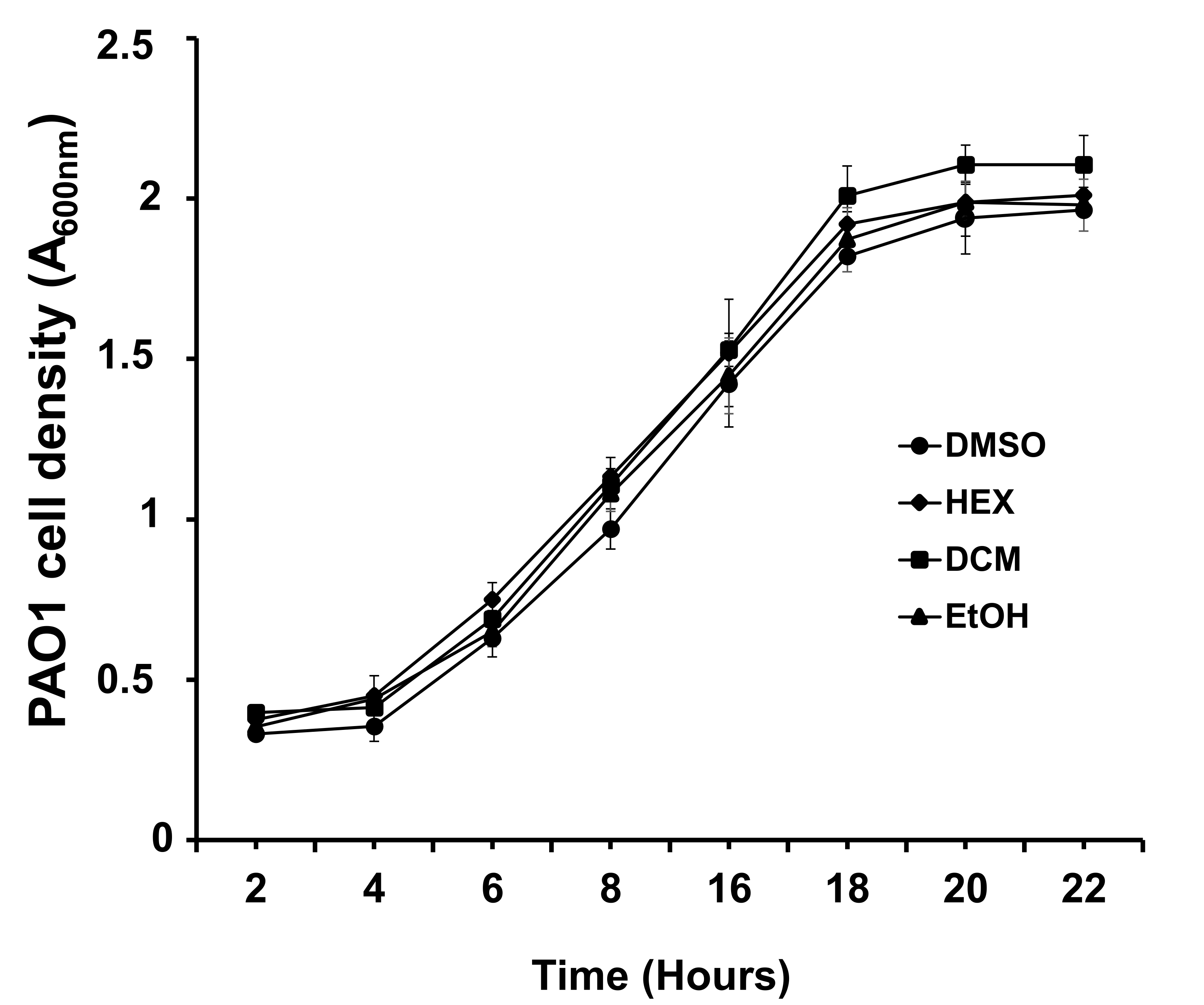

Supplement: Supplementary file 1 [file plants-13-01122-s001.zip › FIgureS1.tif]

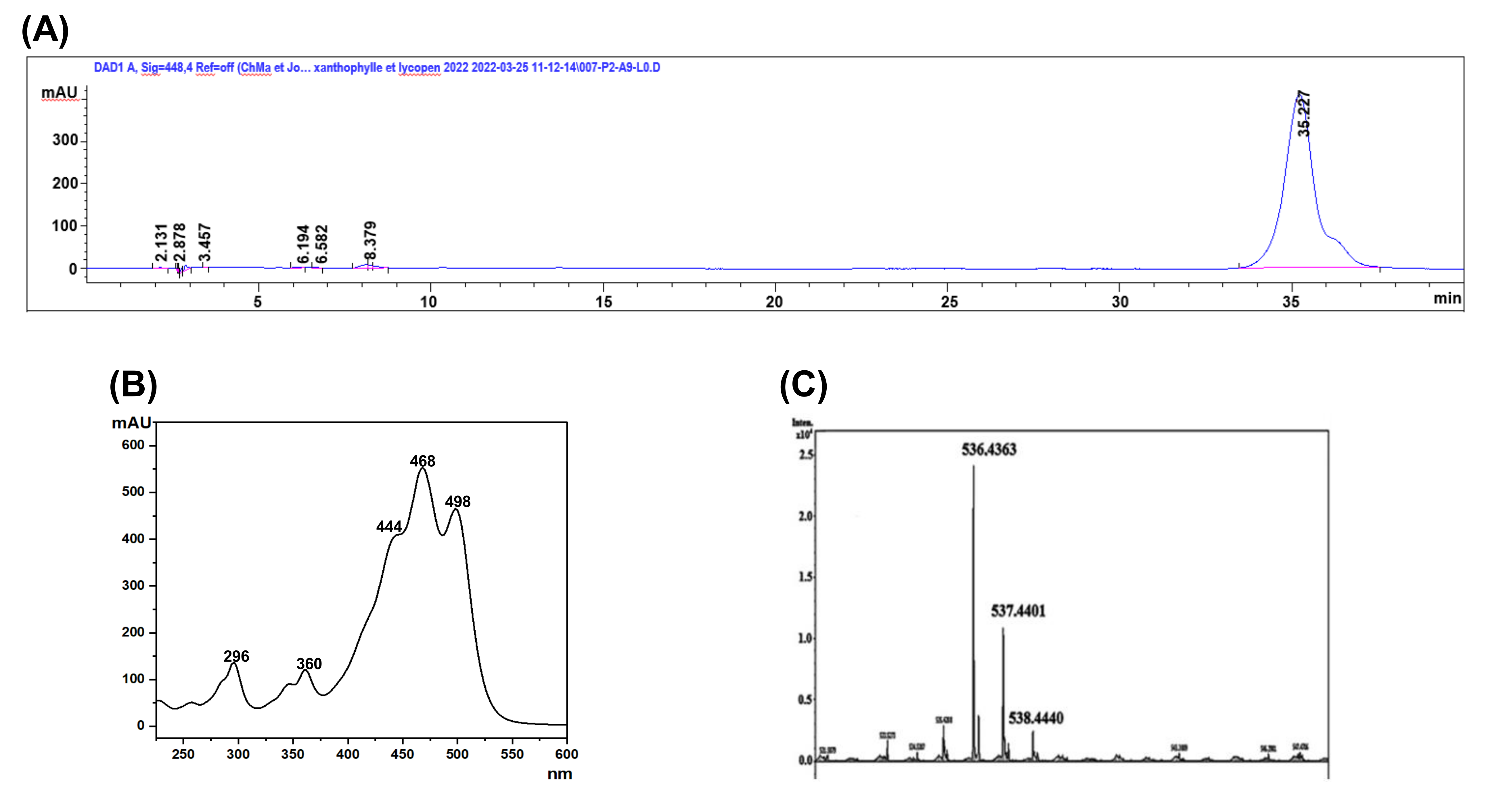

Supplement: Supplementary file 1 [file plants-13-01122-s001.zip › FigureS3.tif]

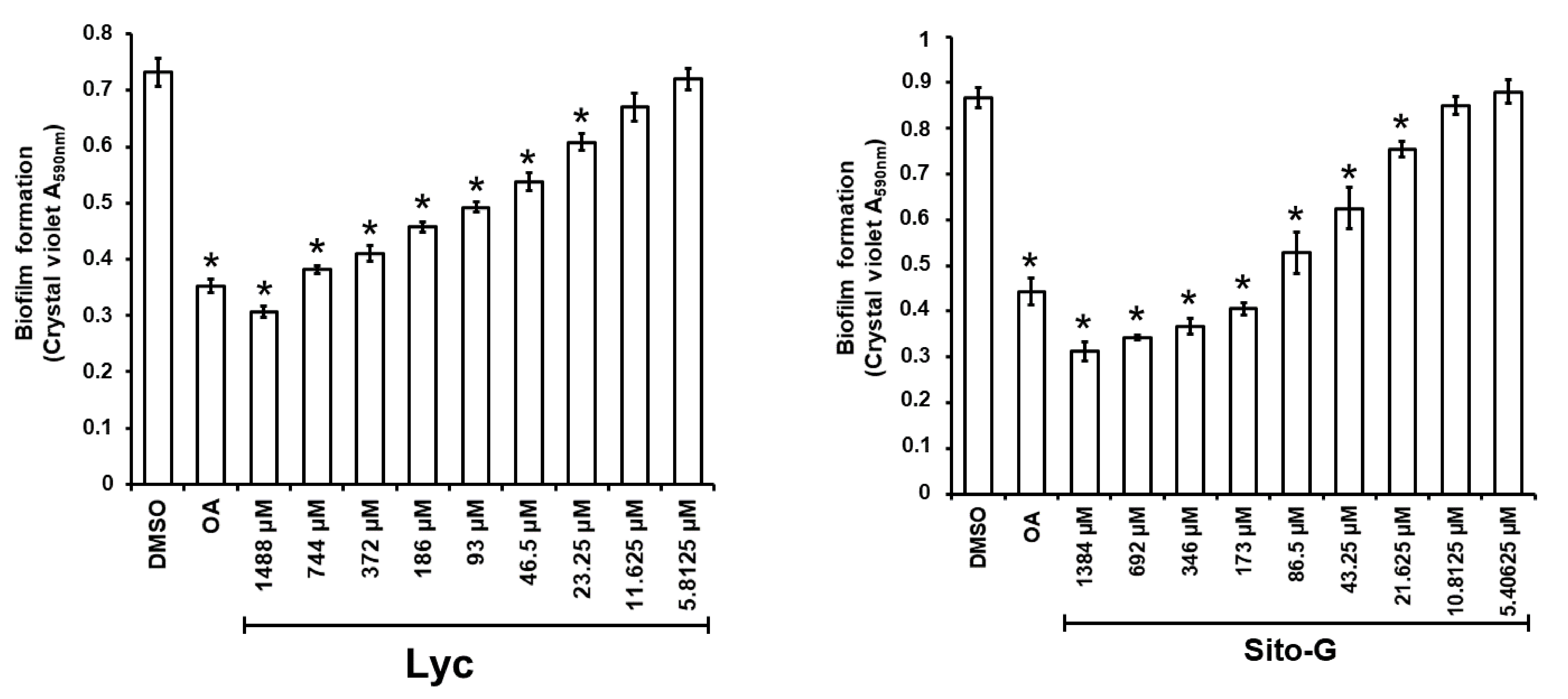

Supplement: Supplementary file 1 [file plants-13-01122-s001.zip › FigureS4.tif]

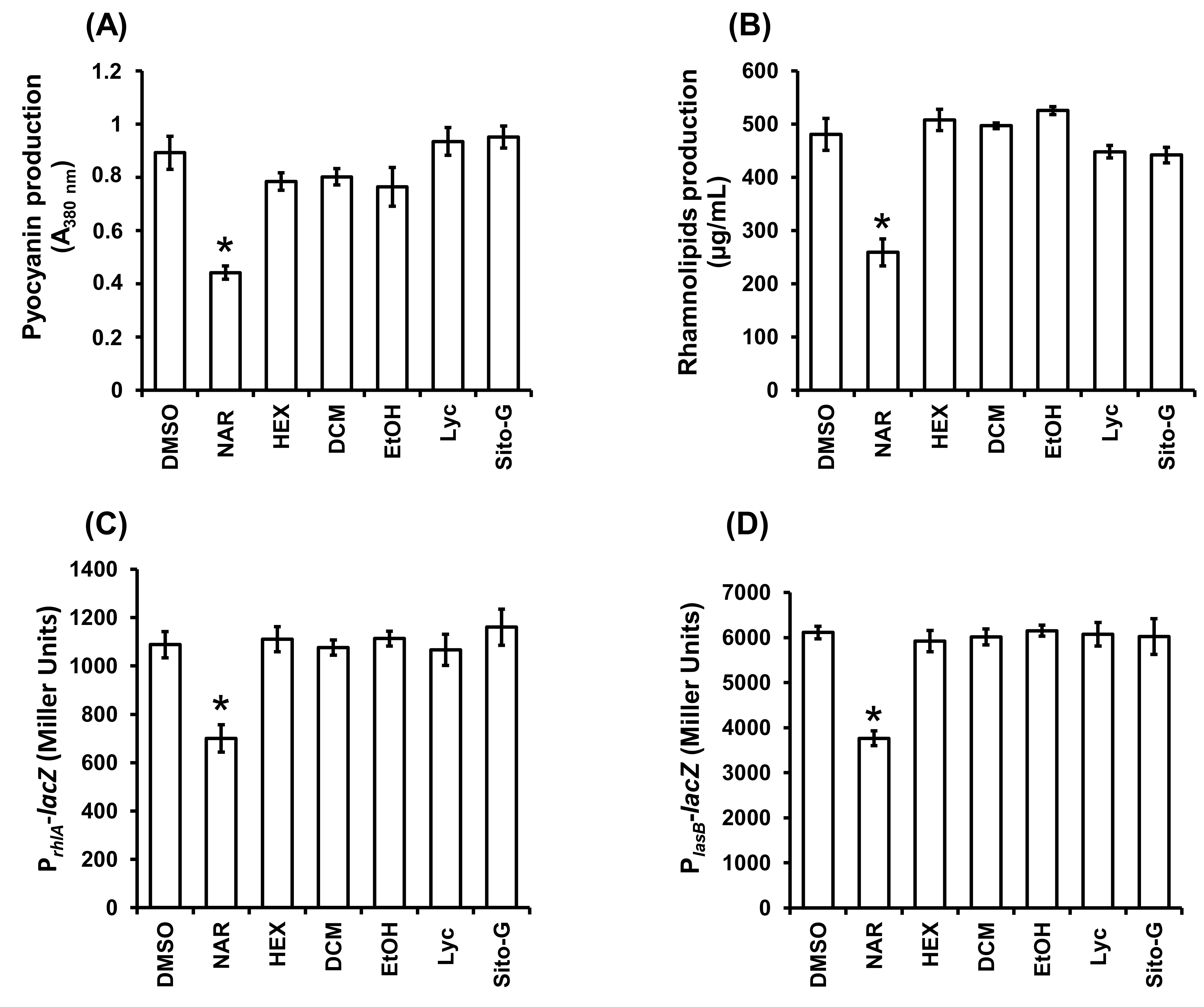

Supplement: Supplementary file 1 [file plants-13-01122-s001.zip › FigureS5.tif]
